# Periodontal Disease and Breast Cancer: A Meta-Analysis of 1,73,162 Participants

**DOI:** 10.3389/fonc.2018.00601

**Published:** 2018-12-12

**Authors:** Jun Shao, Lan Wu, Wei-Dong Leng, Cheng Fang, You-Jia Zhu, Ying-Hui Jin, Xian-Tao Zeng

**Affiliations:** ^1^Department of Stomatology, Guangzhou Hospital of Integrated Traditional and West Medicine, Guangzhou, China; ^2^Center for Evidence-Based and Translational Medicine, Zhongnan Hospital of Wuhan University, Wuhan, China; ^3^Department of Evidence-Based Medicine and Clinical Epidemiology, The Second Clinical College of Wuhan University, Wuhan, China; ^4^Department of Stomatology, Zhongnan Hospital of Wuhan University, Wuhan, China; ^5^Department of Stomatology, Taihe Hospital, Hubei University of Medicine, Shiyan, China

**Keywords:** periodontal disease, breast cancer, risk factor, periodontal therapy, meta-analysis

## Abstract

**Objective:** To investigate the correlation between periodontal disease and breast cancer.

**Materials and Methods:** PubMed and China National Knowledge Infrastructure (CNKI) databases were searched up to February 8, 2018 for observational studies examining the association between periodontal disease and breast cancer. Study selection was conducted according to predesigned eligibility criteria, and two authors independently extracted data from included studies. Meta-analysis was performed using the Comprehensive Meta-Analysis v2 software and risk estimates were calculated as relative risks (RRs) with corresponding 95% confidence intervals (CIs).

**Results:** A total of 11 study were included. Meta-analysis indicated that periodontal disease significantly increased the risk of breast cancer by 1.22-fold (RR = 1.22, 95% CI = 1.06–1.40). Amongst participants with periodontal patients and a history of periodontal therapy, the risk of developing breast cancer was not significant (RR = 1.23; 95% CI = 0.95–1.60). The association results between periodontal diseases and breast cancer were found to be robust, as evident in the leave-one-out sensitivity analysis.

**Conclusions:** Periodontal disease may be a potential risk factor for the development of breast cancer among women, and thus effective periodontal therapy may present as a valuable preventive measure against breast cancer.

## Introduction

Periodontal disease, a complex polymicrobial inflammatory disease, is a public health burden and is partially responsible for tooth loss. Existing epidemiological studies have linked periodontal disease to numerous systemic conditions, such as cardiovascular disease (1), preterm birth (2), osteoporosis (3), and diabetes mellitus (4), all of which may be attributed to systemic infection and inflammation (5). In recent years, there is increased interests in exploring the relationship between periodontal disease and cancer risk, particularly for cancers in the head and neck, upper gastrointestinal system, lung, and pancreas (6–9).

Cancer is a leading cause of mortality worldwide. Among the female-specific malignancies, breast cancer remains the most common cancer type in developed countries, accounting for one-third of newly diagnosed cancers (10). Available data indicated a number of risk factor associated with this cancer, such as endogenous hormone levels, immune factors and genetic susceptibility and lifestyle habits (11–14). Periodontal health status has also been identified as a risk factor for breast cancer, a study of Chung et al. (15), reported an increased risk of breast cancer among patients with chronic periodontitis [Hazard ratio (HR) = 1.23, 95% confidence interval (CI) = 1.11–1.36] although no such observation was found amongst subjects who underwent gingivectomy or periodontal flap operation (HR = 1.17, 95%CI = 0.86–1.58). However, Published studies reported inconsistent findings on the association between periodontal disease and breast cancer, with some supporting their significant association while others claiming none. For example, Freudenheim et al. (16), reported that periodontal disease significantly increased breast cancer risk (HR = 1.14, 95% CI = 1.03–1.26), particularly among former smokers who quit within 20 years (HR = 1.36, 95%CI = 1.05–1.77); while Mai et al. (17) and Han et al. (18) reported different observations. Given that single epidemiologic studies may be inadequate for identifying etiological influence of periodontal disease on breast cancer. We consequently conducted this meta-analysis of case-control and cohort studies to provide updated evidence on the relationship between periodontal disease and female-specific breast cancer.

## Materials and Methods

Our study was conducted and reported according to the Preferred Reporting Items for Systematic Reviews and Meta-Analyses (PRISMA) statement (19).

### Eligible Criteria

Studies were eligible for inclusion if they met the following criteria: (1) adopting a cohort or case-control design; (2) study participants with periodontal disease, as the exposure of interest; (3) female-specific breast cancer, as the outcome of interest; (4) providing relative risks (RRs), HRs or odds ratios (ORs) and corresponding 95% CIs, or data for their calculation; (5) published in full in English-language or Chinese-language peer reviewed journals. For multiple study reports with the same study population, data from the longest follow-up duration were extracted and analyzed.

### Search Strategy

PubMed and China National Knowledge Infrastructure (CNKI) databases were searched up to February 8, 2018 for case-control and cohort studies examining the association between periodontal disease and breast cancer. The following key words and subject terms were used: “periodontitis,” “periodontal disease,” “oral status,” “dental health,” “breast cancer,” “cancer,” and “carcinoma.”

### Data Extraction

Two authors independently extract the following data extraction from all eligible studies: the first author's name, year of publication, country, study design, follow-up period, age, sample size, periodontal disease, and breast cancer ascertainment, type of periodontal therapy, reference of control, dental and smoking status, risk estimates with corresponding 95%CIs, and corresponding adjustments. Any disagreements over the extracted data were resolved by consensus or by consulting a third author.

### Statistical Analysis

Association between periodontal disease and breast cancer risk was assessed using risk ratios (RRs) and corresponding 95%CIs across the included studies. HRs and incidence density ratios (IDRs) were directly considered to be equivalent to RRs. Where ORs were reported they were transformed into RRs using this this formula: RR = OR/[(1 – *P*_0_) + (*P*_0_ × OR)], in which *P*_0_ is the incidence of the outcome of interest in the non-exposed group (20), standard errors (SEs) of the corrected RRs were calculated from this formula: SElog (relative risk) = SElog (odds ratio) × log(relative risk)/log(odds ratio) (21). When these data could not be obtained through calculation, the ORs were directly pooled with tolerance. Since these transformations may underestimate the variance of the RRs derived from the ORs (22, 23), we performed a sensitivity analysis by excluding studies for which this transformation had been applied. For two studies (17, 24) that reported HRs for mild/moderate periodontitis and severe periodontitis, respectively, we constructed subgroups and calculated a combined HR using a fixed-effect model for the main analysis. Heterogeneity among included studies was investigated using the *I*^2^ statistics and *Q* test, and low statistical heterogeneity was defined as *I*^2^ ≤ 50% and *p* > 0.1, respectively, A fixed-effects model was utilized in analyses where statistical heterogeneity was shown to be low; otherwise a random-effect model was adopted (25). Subgroup analyses were conducted according to different characteristics of the included studies. The leave-one-out sensitivity analysis was performed to detect the robustness of the association results by removing one study at a time, Publication bias among the included studies was examined visually using funnel plots (26). Statistical analyses were undertaken using the Comprehensive Meta-Analysis v2.2 software (CMA v2.2) (27).

## Results

### Study Selection and Characteristics

**Figure 1:**
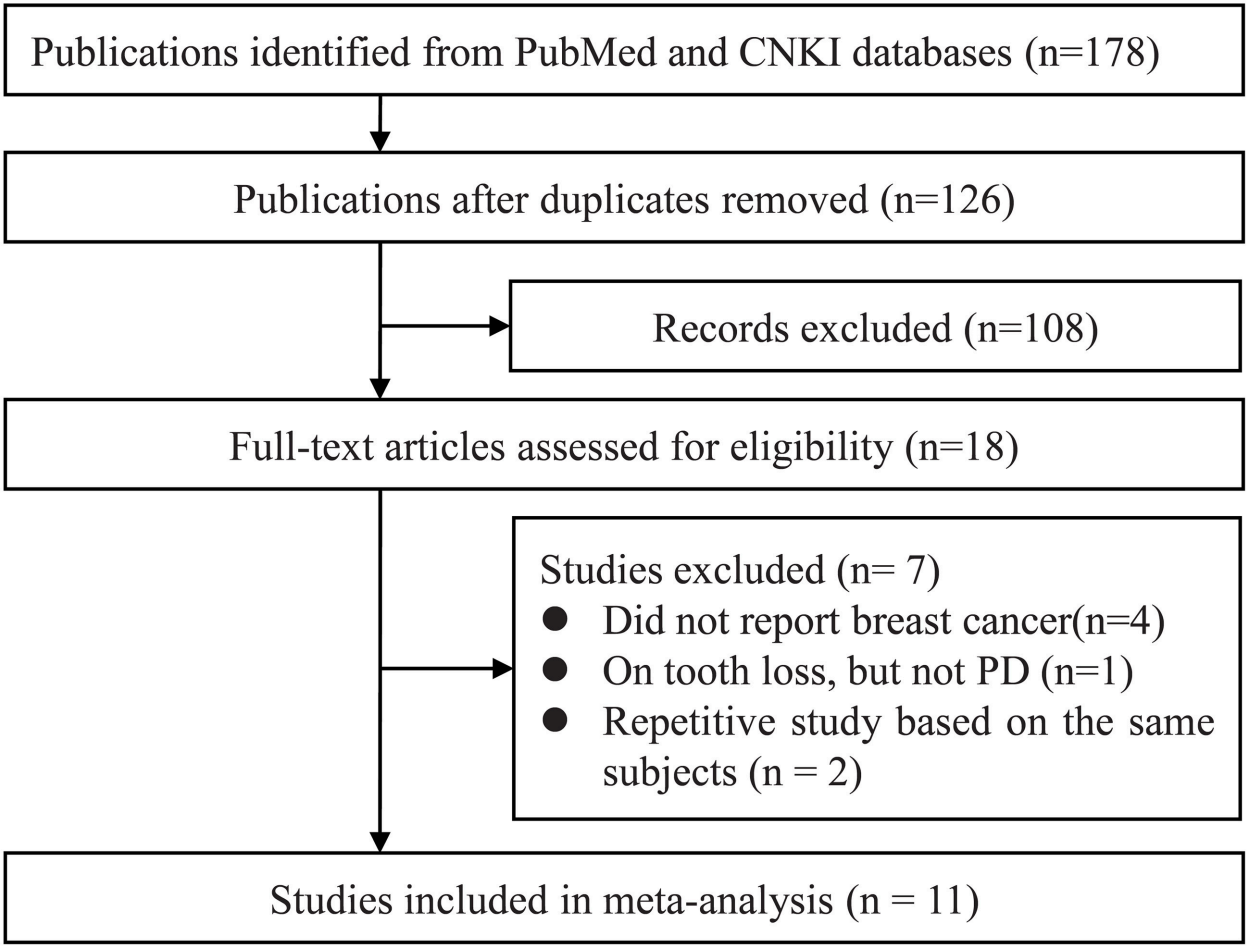
Study selection process. CNKI, China National Knowledge Infrastructure; PD, periodontal disease.

Our systematic literature search yielded a total of 178 publications. After de-duplication 126 titles and abstracts were assessed for eligibility, of these, full texts articles of 18 records were retrieved foe further assessment. Eventually, 11 studies were included in the qualitative synthesis (15, 17, 24, 28–34) (Figure 1).

The 11 included studies were published in the period of 2003 and 2018, Of these, 7 were prospective cohort studies, 3 were retrospective cohort studies and one was case-control study, involving a total of 1,73,162 of whom 3,953 were diagnosed with breast cancer. Among these studies, one focused on co-twin (29), two on postmenopausal woman (17, 32) and on individuals who had a history of periodontal therapy and on females who are currently treated by periodontal therapy (15, 31, 34). Three included studies (15, 31, 32) also provided information on the participants′ baseline dental or smoking status. Majority of the included studies (15, 17, 24, 28–34) chose healthy subjects without periodontal disease as controls, however one study (31) enrolled national cancer registry figures as the control group. All of studies performed covariate adjustment. Characteristics of included studies are summarized in Tables 1, 2.

**Table 1:** Characteristics of included studies.

**Study ID**	**Sample**	**Study design (study period)**	**Follow-up period (yrs)**	**Age (yrs)**	**Cases/ sample**	**PD ascertainment**	**Breast cancer ascertainment**	**Periodontal therapy**	**Control**	**Dental /smoking status**	**Risk estimates (95% CI)**
Hujoel et al. (28)	The NHANES I Epidemiologic Follow-up Study (NHEFS); USA	Prospective cohort (1971/1975–1992)	10	25–74	79/6862	Clinical diagnosis	ICD-9	Unreported	No PD	PD	OR = 1.32 (0.74–2.38)
										Gingivitis	OR = 0.62 (0.32–1.21)
										Edentulism	OR = 0.59 (0.32–1.10)
Arora et al. (29)	The Swedish Twin Registry; Sweden	Prospective cohort (1963–2004)	27	38–77	531/8433	Self-report	ICD	Unreported	No PD	PD	HR = 1.12(0.75–1.68)
										Minor mobility	HR = 1.22(0.92–1.61)
Söder et al. (30)	A registry database of all inhabitants of Stockholm County; Sweden	Prospective cohort (1985–2001)	16	30–40	39/1586	Clinical diagnosis	ICD-7, ICD-9, ICD-10	Unreported	No PD	PD	OR = 13.08 (3.09–55.32)
Chung et al. (15)	The Taiwan National Health Insurance (NHI) program; China	Retrospect cohort (1995–2004)	5	54.1 ± 11.5	367/42548	Clinical diagnosis	ICD-9-CM	Included	No PD	CP	HR = 1.23(1.11–1.36)
										Periodontal therapy	HR = 1.17(0.86–1.58)
Mai et al. (17)	The Buffalo OsteoPerio Study; USA	Prospective cohort (1997–2014)	12.2 ± 4.2	53–85^*^	89/1337	Measured ACH	Medical records	Unreported	No PD	Mild/moderate P	HR = 1.15 (0.67–1.99)
										Severe P	HR = 0.94 (0.49–1.82)
										Over all^#^	HR = 1.06 (0.70–1.61)^#^
Dizdar et al. (31)	The Hacettepe University Dentistry and Oncology hospitals in Ankara; Turkey	Retrospect cohort (2001–2010)	12	49.6	5/151	Clinical and radiographic parameters	Patient files and hospital registries	Included	No control, use national cancer registry figures	CP (moderate or severe)	RR = 2.40 (0.88–6.55)
Han (18)	The sixth Korean National Health and Nutrition Examination Survey (KNHANES); Korea	Prospective cohort (2013–2015)	2	>19	84/1979	Community periodontal index	Health interview	Unreported	No PD	PD	OR = 1.56(0.79–3.06)
Nwizu et al. (32)	Women's Health Initiative Observational Study (WHI-OS); USA	Prospective cohort (1994/1998–2013)	8.32	50–79^*^	2416/65869	Self-report and medical records	ICD-O-2	Unreported	No PD	PD	HR = 1.13 (1.03–1.23)
										Never smokers	1.05 (0.92–1.21)
										Former smokers	1.22 (1.08–1.37)
										Current smokers	1.32 (0.89–1.97)
Sfreddo et al. (33)	The University Hospital of Santa Maria (UHSM); Brazil	Case-control (2013–2015)	/	55.0 ± 8.9/ 54.6 ± 8.7	67/201	Clinical diagnosis	ICD-10 C50	Unreported	No PD	PD	OR = 2.72(1.18-6.27) Corrected RR = 2.07(1.13–3.80)
										Never smokers	OR = 5.08(1.66–15.61)
										Non-drinkers	OR = 2.39(1.03-5.52)
Heikkila et al. (34)	The patient register of the Public Dental Service of the City of Helsinki; Finland	Prospective cohort (2001/2002–2013)	10.1	>29	74/40108	periodontal pocket depth	ICD-10	Included	No PD	PD	RR = 1.19 (0.66–2.12)
Michaud et al. (24)	The Atherosclerosis Risk in Communities (ARIC) study; USA	Prospective cohort (1987–2012)	14.7	44–66	202/4088	Clinical diagnosis	Patient files and hospital registries	Unreported	No PD	Mild	HR = 1.32(0.81–2.14)
										Moderate	HR = 0.89(0.61–1.29)
										Severe	HR = 0.84(0.49–1.43)
										Over all#	HR = 0.98(0.76–1.27)
										Edentulism	HR = 0.88(0.56–1.36)

* Postmenopausal women; **#**Over all data was calculated by pooling mild/moderate P and severe P using a fixed-effects model for the final analysis.

*ACH, Oral alveolar crestal height; ICD, International classification of diseases; CP, chronic periodontitis; PD, periodontal disease; P, Periodontitis; CI, Confidence interval; HR, Hazard ratio; OR, Odds ratio; RR, Relative risk*.

**Table 2:** Covariate adjustments performed in the included studies.

**Study ID**	**Adjusted covariates**
Hujoel et al. (28)	Age
Arora et al. (29)	Age, education, employment, number of siblings, smoking status, smoking status of partner, alcohol status, and body mass index
Söder et al. (30)	Age, education level, socio economic status, working history, yearly income, smoking in pack-years, dental appointments, dental plaque index, gingival bleeding index, and loss of any molar tooth in the mandible
Chung et al. (15)	Patient's monthly income, geographic region, and diabetes
Mai et al. (17)	Age at visit (continuous) and pack-years of smoking (continuous)
Dizdar et al. (31)	Age
Han (18)	Household income, education, smoking status, drinking frequency, and chronic disease
Nwizu et al. (32)	Age, BMI
Sfreddo et al. (33)	Age, parity, total breastfeeding time, hormonal replacement therapy, BMI
Heikkila et al. (34)	Calendar time, age, socio-economic status, number of teeth, dental treatments (gingivitis, caries, endodontic caries, surgery and prosthesis), oral health indices (I, D, DMF), need of periodontal treatment and diabetes
Michaud et al. (24)	Age, field center, education level, smoking status, smoking duration, drinking status, body mass index, and diabetes status (diagnosed diabetes, undiagnosed diabetes, at risk for diabetes [reference is normal]), hormone replacement therapy use, joint terms for field center and race

*BMI, Body mass index; I, Initial caries; D, Decayed teeth; DMF, Decayed-missing-filled teeth; NSAID, Non-steroidal anti-inflammatory drug*.

### Overall Analysis

**Figure 2:**
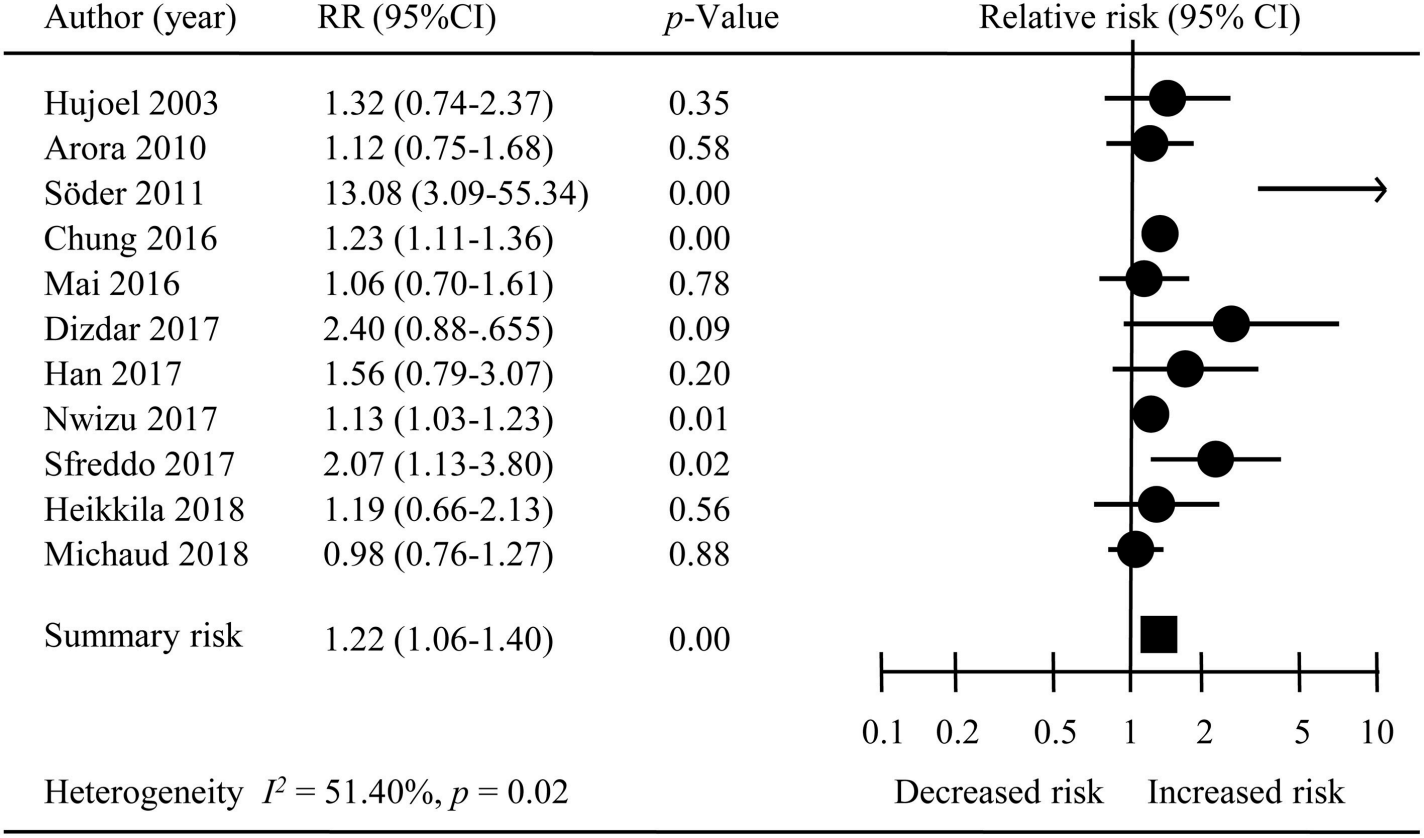
The overall result of meta-analysis. RR, Relative risk; CI, Confidence interval.

**Figure 3:**
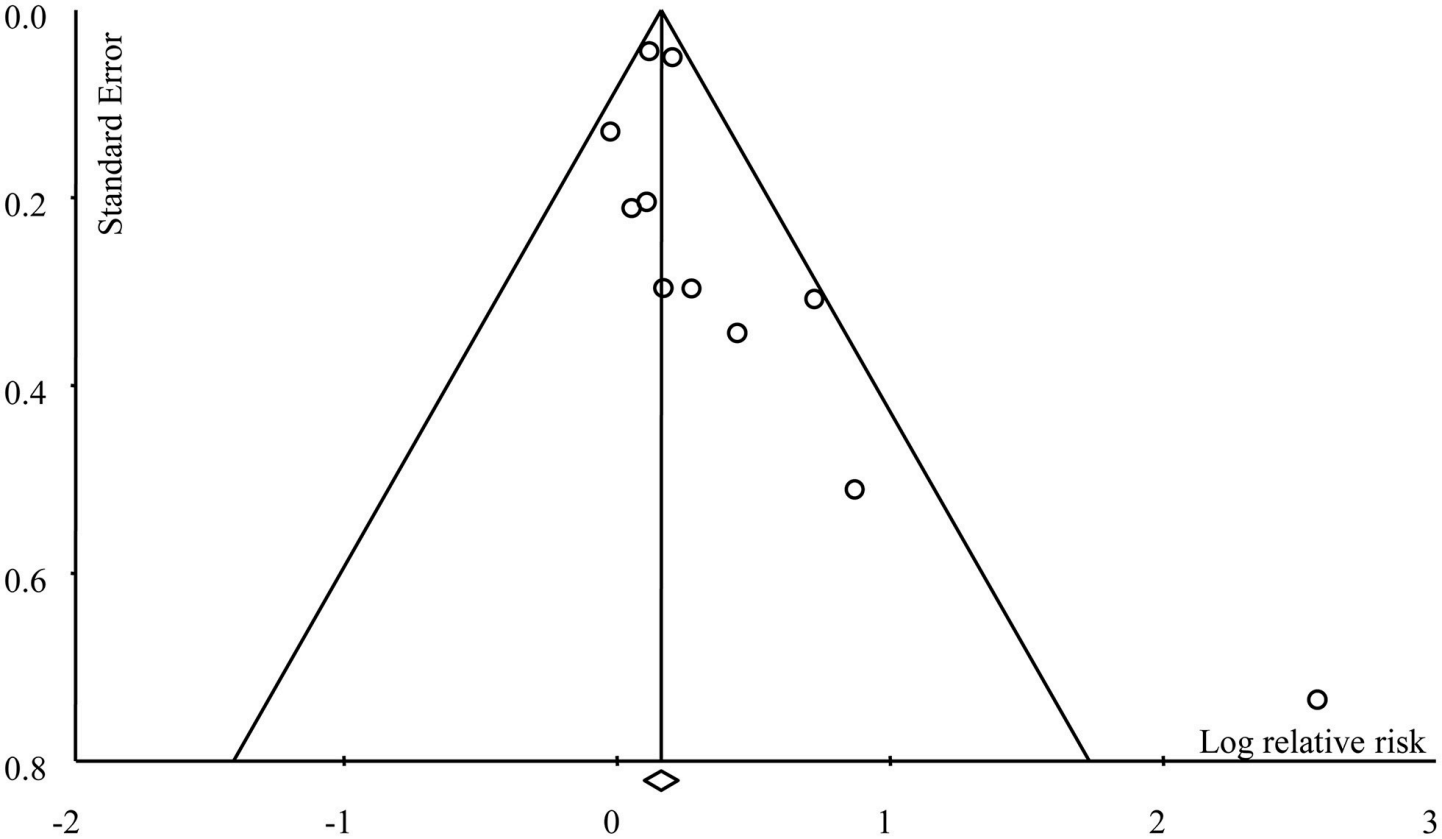
Funnel plot for publication bias.

The overall meta-analysis of 11 studies suggested that the risk of developing female-specific breast cancer was 1.22 times greater amongst patients with periodontal disease than those without periodontal disease (RR = 1.22, 95% CI = 1.06–1.40; Figure 2). Moderate statistical heterogeneity was noted across the 11 included studies (*I*^2^ = 51.40 %; *P* = 0.04). Visual inspection of a funnel plot revealed a degree of publication bias (Figure 3).

### Subgroup Analyses

A number of pre-specified subgroup analyses, including study design, follow-up period, pausimenia status, medical history of periodontal therapy, and dental status were performed (Table 3). The impact of study design on final results was investigated by stratifying analyses according to prospective cohort study design (*n* = 8) (17, 18, 24, 28–30, 32, 34), retrospect cohort study design (*n* = 2) (15, 31) and case-control study design (33), and the results showed statistical difference (Prospective cohort: RR = 1.17, 95% CI = 0.97–1.41; retrospective cohort: RR = 1.24, 95% CI = 1.12–1.37). In terms of follow-up duration, three studies (15, 18, 32) that followed study participants for < 10-year indicated a significant impact of periodontal disease on the risk of developing breast cancer (RR = 1.18, 95% CI = 1.10–1.26); in the 7 studies (17, 24, 29–34) with follow-up period of ≥10 years, the association of periodontal disease and breast cancer risk was not statistically significant (RR = 1.45, 95% CI = 0.95–1.76). And these results suggested that study design and follow-up period might have certain effect on study results. Amongst three studies describing the use of periodontal therapy (15, 31, 34), results of subgroup analysis indicated a non-significant association of periodontal disease and breast cancer (RR = 1.23; 95% CI = 0.95–1.60); analysis of eight studies (17, 18, 24, 28–30, 32, 33) without clear reporting of revealed a distinct relationship of periodontal disease and breast cancer risk (RR = 1.25, 95% CI = 1.01–1.55). Results of the subgroup analyses according to pausimenia status and dental status were consistent with the overall results. Due to limited data, we were unable to pursue subgroup analysis by study participants' smoking status.

**Table 3:** Results of the overall meta-analysis and subgroup analyses.

**Type of analysis**	**No. of studies**	**Heterogeneity**		**Model**	**Results**		
		***p***	***I^**2**^*(%)**		**RR**	**95% CI**	***P*-value**
**Overall analysis**	11	0.04	51.40	Random	1.22	1.06–1.40	<0.01
**SUBGROUP: STUDY DESIGN**							
Prospective cohort	8	0.06	48.22	Random	1.17	0.97–1.41	0.09
Retrospective cohort	2	0.19	40.71	Fixed	1.24	1.12–1.37	<0.01
Case-control	1	-	-	-	2.07	1.13–2.91	0.02
**SUBGROUP: FOLLOW-UP PERIOD**							
<10 years	3	0.33	8.94	Fixed	1.18	1.10–1.26	<0.01
≥10 years	7	0.02	59.49	Random	1.29	0.95–1.76	0.11
**SUBGROUP: MENOPAUSAL STATUS**							
Pausimenia	2	0.77	<0.01	Fixed	1.13	1.03–1.23	<0.01
Unspecified	9	0.02	57.01	Random	1.35	1.08–1.68	0.01
**SUBGROUP: PERIODONTAL THERAPY**							
Included	3	0.40	<0.01	Fixed	1.23	0.95–1.60	0.26
Unreported	8	0.02	59.47	Random	1.25	1.01–1.55	<0.01
**SUBGROUP: DENTAL STATUS**							
CP	2	0.19	40.71	Fixed	1.24	1.12–1.37	<0.01
PD	9	0.03	52.74	Random	1.24	1.02–1.50	0.03
**SUBGROUP: SENSITIVITY ANALYSES**							
Excluded studies results reported as ORs	7	0.48	<0.01	Fixed	1.16	1.09–1.23	<0.01
Excluded studies based on co-twin	10	0.01	56.14	Random	1.23	1.06-1.43	0.09
Excluded studies with controls with PD	10	0.03	51.68	Random	1.20	1.05–1.37	0.01

*CP, Chronic periodontitis; PD, Periodontal disease; OR, Odds ratio; RR, Relative risk; CI, Confidence interval*.

### Sensitivity Analyses

**Figure 4:**
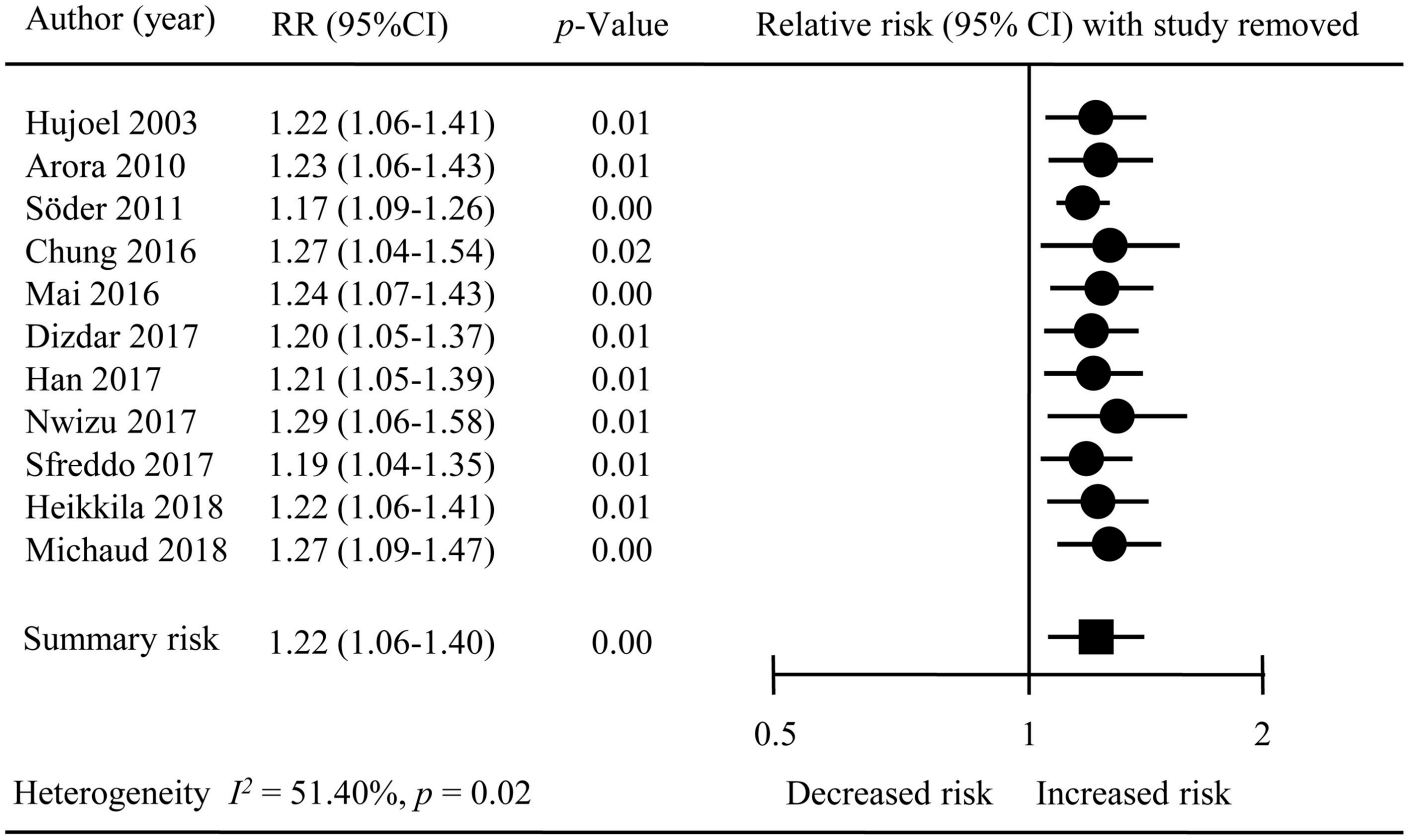
The result of sensitivity analysis.

We conducted sensitivity analysis by removing one study at a time from the overall met-analysis (Figure 4), Omission of a study (31), which did not precisely state whether its control individuals had periodontal disease, did not lead to any substantial changes in the overall results (RR = 1.20; 95% CI = 1.05–1.37); similar observation was noted upon exclusion of a study on co-twin (29) (RR = 1.23; 95% CI = 1.06–1.43). We also sequentially removed four studies (18, 28, 30, 33) reporting their association results, and a noticeable change in statistical heterogeneity was observed, (overall analysis: *I*^2^ = 51.40 %, *P* = 0.04;sensitivity analysis: *I*^2^ = < 0.01, *P* = 0.48) although the pooled estimates (RR = 1.16; 95% CI = 1.09–1.23) remained consistent between the overall results.

The leave-one-out sensitivity analysis led to a decline of the level of statistical heterogeneity declined from moderate (*I*^2^ = 51.40 %, *P* = 0.04)to low one (*I*^2^ = 8.56%, *P* = 0.36) upon removing the study by Söder et al. (30), indicating that this study contributed to a considerable to the statistical part of heterogeneity across studies.

## Discussion

This meta-analysis of 11 observational studies suggested that periodontal disease might be a risk factor for female-specific breast cancer. Finding from sensitivity analyses confirmed the robustness of our association results. According to the findings from subgroup analyses, a number of confounding factors might be influencing the results of the overall analysis, including study design, follow-up period, and history of periodontal therapy.

Periodontal disease is a complex infectious diseases (35), and its association with cancer risk has attracted increasing attentions over the years (36, 37). Existing research provided useful insights on the role of periodontal disease treatment in reducing the risk of different types of cancers (38). Possible mechanisms behind the association between periodontal disease and cancers have been proposed, and the most widely advocated theory is the alteration of the oral flora and subsequent influx of the inflammatory markers into the systemic circulation (35, 39). Numerous studies suggested that periodontal disease might be a potential risk factor for cancers in the oral, digestive, respiratory and reproductive systems (6–9, 40, 41). Moreover, existing data revealed a prevalence of periodontal disease among females during different time periods, including puberty, menstruation, pregnancy, and menopause (42–44); and periodontal disease in women has been found to be positively associated with autoimmune diseases, disorders infertility, adverse pregnancy, low birth weight, and preterm birth, and breast cancer (45, 46). Consequently, it has been suggested that oral health amongst females should be a cause for concern.

It is worth highlighting that a similar meta-analysis by Shi et al. (47) was published recently. Though this meta-analysis was conducted with sound methodology and reported clearly according to validated guidelines, our current meta-analysis has a number of strengths. First, the mentioned meta-analysis (47) studies whereas our findings were based on a large evidence base of 11 observational studies. This is due to a more comprehensive search period and study selection approach. Second, Shi et al. (47) only investigated the association between periodontal disease and breast cancer; however, we also specifically investigated the influence of confounding factors such as pausimenia status and history of periodontal therapy on the association between periodontal disease and breast cancer risk. Third, in regards to the calculation of risk estimates, Shi et al. (47) considered HRs/ORs as RRs, whereas we took an extra step to transform ORs into RRs, thereby providing more reliable implications for clinical practice and further researches.

Despite our efforts, however, there are some limitations of our meta-analysis. First, although we were able to identify one (30) contributing to statistical heterogeneity, we were unable to establish the precise causes of such influence. Second, definition of periodontal disease varied between the included studies. Where certain studies used tooth mobility as disease marker, some were self-reported disease status, some used patient medical records for confirmation, and some studies employed clinical or radiographic examinations as final diagnostic status. Third, consideration of confounding factors varied across studies and certain valuable factors such as history of periodontal therapy, baseline smoking status and dental health status, were not consistently reported, and thus findings of the corresponding subgroup analyses remained inconclusive. Fourth, there could be potential control selection bias since we included studies that recruited participants without periodontal disease as controls, as well as one study which adopted national cancer registry figures as the reference (31), although findings from sensitivity analysis revealed robustness of our overall results. Finally, owing to limited data, we were unable to further explore association between various severity of periodontal disease and different subtypes of breast cancer

Based on currently available evidence, we urge healthcare professionals and the general public to be further aware of the potential association between periodontal disease and breast cancer. The current meta-analysis provided useful insights on the potential effect of periodontal disease on the risk of developing breast cancer, however, further research establishing the precise mechanisms underlying such effect, such as the relationship between altered oral flora, inflammatory marker influx and hormonal levels is warranted. Furthermore, the influences of potential confounding factors, such as smoking status, dietary history, use of contraceptives, marriage, and childbirth status, stress, types of breast cancer, severity of periodontal disease should also be explored.

## Conclusion

This meta-analysis indicated that periodontal disease might be a potential risk factor for the development of breast cancer in women, and effective periodontal therapy could pose as a viable preventive measure against breast cancer. However, the association between different severity of periodontal disease and sub-types of breast cancer remain to be ascertained. Nonetheless, we are confident that the findings of the current meta-analysis could be helpful in increasing awareness and importance of oral health maintenance, which may led to a reduced risk of developing breast cancer.

## Author Contributions

JS, LW, and X-TZ designed this study. CF and Y-HJ performed search and collected data. Y-JZ re-checked data. LW, CF, and Y-HJ performed analysis. JS and LW wrote the manuscript. X-TZ reviewed the manuscript.

### Conflict of Interest Statement

The authors declare that the research was conducted in the absence of any commercial or financial relationships that could be construed as a potential conflict of interest.
